# Colloid stability of iron compounds in groundwater of Western Siberia

**DOI:** 10.1186/2193-1801-3-260

**Published:** 2014-05-22

**Authors:** Ludmila N Shiyan, Elena А Tropina, Ksenia I Machekhina, Elena N Gryaznova, Vladimir V An

**Affiliations:** Laboratory 12, Institute of High Technology Physics, Tomsk Polytechnic University, Tomsk, Russia; 30, Lenin Ave, 634050 Tomsk, Russia

**Keywords:** Groundwater, Colloid solutions of iron compounds, Experimental modelling, Coagulation stability, Colloid solution stability

## Abstract

The paper reports on experimental modeling of the colloid system composition in natural groundwater. Iron hydroxide is found to be the main component of natural colloid systems. It is shown that silicon compounds and dissolved organic substances (DOS) stabilize iron hydroxide (III), forming a stable colloid system, and preclude coagulation. The presented results suggest that CaCl_2_ and AlCl_3_ electrolytes affect the coagulation stability of synthesized model colloid solutions.

## Introduction

Now, colloid chemistry faces problems of interlinking theoretical knowledge and experimental approaches and preparing colloid systems for use in different industries. Colloid systems are used to increase productivity in chemical industry and technology, agriculture and animal industry. They are also used to manufacture highly effective pharmaceutical products. The most advanced technologies of substance dispersion and colloid solution stabilization are employed to obtain colloid systems. Various types of colloid systems exist in the nature or arise spontaneously in industrial and natural processes, e.g., on extraction and purification of natural waters. This is due to high stability of colloid solutions based on iron compounds. Colloids of iron compounds are found in natural waters all round the world. It is shown that humic-type organic substances play an important role in the formation of iron compounds (Salanko et al. 2007; Perminova et al. 2003). In Russia, where boggy areas occupy half the country and iron ions are present almost in all water sources, the problem of colloid removal is of immediate interest.

Despite the abundance of open waters in Western Siberia, groundwater remains to be the main drinking water supply on this territory (Zektser and Yasvin 1996). The impurity content in groundwater differs in quantitative composition and qualitative correlation. The main impurities that affect the quality of groundwater in the region and create problems in water preparation are iron compounds in the colloid state. The groundwater of Western Siberia abounds in these compounds due to leaching and dissolution of ferrous minerals, in which the territory is rich (Shvartsev 1996). Marshes and small rivers, which contain humic-type substances, are most likely to contribute to the formation of stable colloid iron compounds.

Developing new and efficient ways of water purification requires data on the impurity properties: atomic, molecular and phase compositions, structure of molecules and colloid particles, and their interaction with water. Direct determination of these properties is often impossible or inefficient due to composition complexity, and this does not allow their correlation with properties of colloid systems. In this context, it is appropriate to study an experimental model of colloid systems and to compare it with natural systems.

The main parameter which can help to estimate the efficiency of different methods of water purification from colloid impurities is coagulation stability. This parameter is more accurately determined by the coagulation rate constant *k*, though the sediment appearance (coagulation time *τ*_c_) depending on the constant is often used to advantage.

For bimolecular coagulation, the initial colloid particle concentration *С*_0_, the coagulation time *τ*_c_, and the coagulation rate constant *k* are related as

where *α = С*_c_/*С*_0_ is the coagulation degree corresponding to the particle concentration *С*_c_ at which the sediment appearance is observed.

The coagulation stability of hydrosols can also be described by the electrolyte concentration *γ*_Е_ at which a sediment appears or the so-called coagulation threshold (Eremenko et al. 1989; Shchukin et al. 2001). Coagulation by electrolytes is associated with hydrosol stability which is normally conditioned by mutual repulsion of charged colloid particles. The particle charge arises due to the difference in cation and anion adsorption energies with attendant formation of a electrical double layer (EDL) characterized by *ζ*-potential. Electrolyte ions (Na^+^, Ca^2+^, Cl^-^, SO_4_^2-^…) decrease the particle charge and potential, thus decreasing the colloid stability due to charge neutralization and EDL compression.

For example, increasing the charge of an ion increases its action on the EDL, and if we add singly, doubly, and triply charged ions to a colloid solution, the calculated coagulation threshold *γ*_Е_ is bound to change in a ratio of 1.00:0.016:0.0013 (Richard 2004). However, experimental values of the coagulation threshold differ greatly from the above values, because colloid solution stability (including that of hydrosols) can be governed, along with EDL formation, by the formation and properties of thin films (solvent layers, various surfactants, and reaction solids) on the particle surface. The presence of several impurities increases the possibility of various interactions, including chemical interactions. In this case, there is no direct connection between coagulation stability, particle charge, and ions in the solutions.

The objective of the work is to synthesize iron compound-based colloid systems close to natural systems in content, to study chemical properties of colloids, and to determine the factors responsible for their coagulation stability.

## Experimental approach

For synthesis and analytical study, reagents FeSO_4_ · 7H_2_O, Na_2_SiO_3_ · 9H_2_O and distilled water were used. Organic substances for experiments were extracted from peat bogs of the Tomsk region. The concentration of organic substances was determined using an analyzer of dissolved organic carbon (DOC) with subsequent IR-analysis of CO_2_ evolved. The initial concentration of organic substances was varied in the range 0.05…4.0 mg/L. The molar mass of organic substances was determined by the gel-chromatography method (Perminova et al. 2003). The samples contained fractions of 200–20000 Da*.*

The iron and silicon concentrations in the solution were determined using an ICP-OES plasma optical emission spectrometer (Varian, USA). The pH value was measured using a WTW Multiline P4 multifunctional device (WTW GmbH, Germany). The colloid particle size distribution and the *ζ*-potential in the model solutions were determined using a Zetasizer Nano ZS analyzer (Malvern Instruments, UK), which allows measuring the particle size in the range 0.6…6000 nm. The particle size was determined by dynamic light dispersion (at a dispersion angle of 173°). The measurement result was in the form of numerical particle distribution s *φ*(*r*) *= dN*/*dr*. The mode *δ*_*m*_ was found from the maximum of this distribution and was taken as an average particle size because the distribution functions approximated normal log functions.

The following combinations of components were chosen for synthesizing the model solutions:

- Solution No. 1 – Iron Compounds (IC);
- Solution No. 2 - Iron Compounds (IC) + dissolved organic substances (DOS);
- Solution No. 3 - IC + silicon compounds (SC);
- Solution No. 4 - IC + DOS + SC.

Stability of the synthesized solutions was estimated from variation in optical density of the solutions, iron colloid concentration in the solutions, *ζ*-potential, and particle size. Simplest model solution No. 1 was prepared by dissolving FeSO_4_ · 7H_2_O in water. The concentration of iron ions in solution No. 1 was 5.6 mg/L and corresponded to the iron concentration in the groundwater of the Western Siberian region. The pH value in the solution was kept at 10.0 ± 0.2 and was taken from measured optimum iron (II) concentration in the solution, sedimentation rate, and system redox potential.

The iron (II) concentration in model colloid solution No. 2 was 5.6 mg/L. The concentration of DOS, which stabilized colloid iron substances, was 0.05…4.0 mg/L. This DOS concentration corresponded to that in the groundwater of the Western Siberian region.

Model colloid solution No. 3 was synthesized with the same iron concentration; the silicon concentration was varied in the range 5…20 mg/L and corresponded to the silicon concentration in the groundwater of the Western Siberian region.

Model solution No. 4 was synthesized with iron (II) ions, DOS, and silicate ions. As shown by analysis of solution No. 2, the particle size in the dispersed phase of the model solution does not depend on the DOS concentration; therefore, the DOS concentration was 4 mg/L. The iron concentration in the solution, like in the others, was constant and was 5.6 mg/L. The silicon concentration was varied in the range 5…20 mg/L.

All model solutions were synthesized at room temperature. A shaker GFL 3005 (Germany) with a shaking frequency 100 rounds/min was used for mixing.

## Results and discussion

### Chemical composition and groundwater indicators in the Western Siberian region

The chemical composition and the groundwater indicators, such as pH value and water color, were measured in the groundwater of Beloyarsky, Kargasoksky, and Strezhevskoy areas and southern areas of the Tomsk region. Table 1 shows the chemical composition and the groundwater indicators in the north and south of Western Siberia.

**Table 1:** **Chemical composition and groundwater indicators of the Western Siberian region**

Indicator	Units	Value		MPC*
		North Western Siberia	South Western Siberia	
рН	-	6.0…7.0	6.8…7.7	6…9
Color index	Grad	30…150	10…45	20
General Iron - Fe(II) + Fe(III)	mg/L	1.0…25.0	0.88…27.0	0.3
Manganese (II)	mg/L	0.03…0.75	0.10…1.35	0.1
General hardness	°H	0.5…6.0	4.5…13.0	7.0
Hydro carbonate - ions	mg/L	30.0…360.0	280.0…800.0	-
Silicon (IV)	mg/L	10.0…28.0	4.5…16.0	10.0
Permanganate index	mgО_2_/L	3.0…14.0	0.9…3.0	5.0
Ratio Са^2+^/Мg^2+^	-	1:1 or 2:1	4:1	-

*The maximum permissible concentration according to the Russian sanitary standards.

The data in Table 1 suggest that the groundwater in the Western Siberia region features high concentration of iron, manganese, silicon, and dissolved organic substances. The iron concentration is typically higher than the maximum permissible concentration (MPC).

It is possible to define impurities that make most critical contribution to the formation of colloid compounds. First, the groundwater contains iron in the form of Fe(II) ions. During oxidation, slightly soluble iron hydroxide (III) appears in the colloid form; the properties of this colloid are well studied (Richard 2004; Paul and Raj 1997). Second, these are humic organic substances which assist the formation of stable iron-containing colloid systems (Serikov et al. 2009). Third, there are silicon compounds which can also contribute to the formation of colloid compounds both with iron compounds and with organic substances (Crittenden et al. 2012).

Thus, we selected iron, silicon, and humic organic substances for synthesizing model solutions and for studying properties of colloid substances in groundwater.

### Synthesis of model colloid solutions

Stability of the synthesized solutions No. 1 was estimated from variation in optical density of the solutions, iron colloid concentration in the solutions, *ζ*-potential, and particle size. It is shown that at a pH value of 10.0 ± 0.2 and Fe^2+^ concentration of 5.6 mg/L, sedimentation occurs in 30 min (*τ*_c_ = 30 min) and the system redox potential changes from -80 to +20 mV. The measurements were taken immediately after preparation of the solution. The results demonstrate that the particle size is larger than 1 μm and the *ζ-*potential is +8 mV. The *ζ-*potential close to zero shows that the colloid system is unstable. Coagulation of colloid iron particles begins and sedimentation occurs because the solubility product for Fe(OH)_3_ is 6.3∙10^-38^ at an iron (III) concentration of about 6.9 · 10^-11^ mol/L. The obtained parameters of the dispersed phase were used to compare solution No. 1 and model solutions No. 2–4.

All solutions No. 2 with a DOS concentration of 0.05…4.0 mg/L are more stable than simplest model solution No. 1. Table 2 shows the DOS concentration determined from the DOC value, particle size, and *ζ* - potential of the dispersed phase for solution No. 2.

**Table 2:** **Properties of the dispersed phase in model solution No. 2**

Iron ion concentration, mg/L	DOS concentration, mg/L	Average particle size, nm	***ζ-***potential, mV	рН
5.6	0.05	91…99	-29	10.0 ± 0.2
	0.25		-32	
	0.5		-38	
	1.0		-38	
	4.0		-42	

The data in Table 2 suggest that the minimum DOS concentration leads to the formation of a protective layer on the surface of colloid particles. Increasing the DOS concentration up to 4 mg/L does nothing to the particle size of the dispersed phase. The average particle size in solution No. 2 is 96 nm, which is less than that in solution No. 1. Charge exchange takes place and the *ζ*-potential acquires a negative charge of –(29…42) mV. It is found that the negative charge of the *ζ*-potential increases with increasing the DOS concentration. This is likely an indication that electrostatic repulsion between like-charged colloid particles affects the formation of a stable colloid system. The obtained solution is stable with time because the iron concentration in the solution remained unchanged for 30 days and was 5.6 mg/L.

Table 3 presents properties of the dispersed phase in model solution No. 3 at different silicon concentrations.

**Table 3:** **Properties of the dispersed phase in model solution No. 3**

Iron ion concentration, mg/L	Silicon ion concentration, mg/L	Average particle size, nm	***ζ-***potential, mV	рН
5.6	5.0	175	-38	10.0 ± 0.2
	10.0	167	-45	
	16.0	78	-48	
	20.0	82	-50	

Colloid iron substances are formed at silicon concentrations of 5.0…20.0 mg/L. However, they partly coagulated at low silicon concentrations such that the particle size increases. The data in Table 3 suggest that at a low silicon concentration of 5.0 mg/L and 10.0 mg/L, the average particle size in the dispersed phase is much higher than that in solution No. 2 and is ~170 nm. As the silicon concentration is increased up to 16.0 and 20 mg/L, the particle size decreases down to 78 and 82 nm. The situation is about the same as that with solution No. 2. If the particles size is less than 170 nm, no coagulation occurs and the solution is stable during 30 days. The *ζ-*potential in solution No. 3, like in solution No. 2, is negative, being equal to -45 mV, and increases slightly with increasing the silicon concentration. This is likely an indication that electrostatic repulsion affects the formation of a stable colloid system.

Table 4 presents properties of synthesized model solution No. 4.

**Table 4:** **Properties of the dispersed phase in model solution No. 4**

Iron ion concentration, mg/L	DOS concentration, mg/L	Average particle size, nm	***ζ-***potential, mV	Silicon ion concentration, mg/L	рН
5.6	4	114	-20	5.0	10.0 ± 0.2
		96	-28	10.0	
		83	-32	16.0	
		92	-29	20.0	

The results obtained in the work suggest that a stable iron colloid solution is formed at a silicon concentration higher than 5 mg/L, because the particle size and the *ζ-*potential depend on the silicon concentration. It is likely that a solid monolayer of silicon compound is formed on the colloid iron particles. At a silicon concentration higher than 15 mg/L, the excess of silicon compounds is in the free state.

Comparison of four model solutions shows that solution No. 1 with no additives is unstable because colloid particles are large and the charge of their surfaces is close to zero at pH =10 ± 0.2. In model solution No. 3, the anions of silicon acid are adsorbed on the surface of colloid iron particles due to low-polarity bonds. This leads to recharging of the particle surface to a negative potential, thus increasing the mutual repulsion of the particles. The particle size decreases and the colloid system stability increases.

A similar result is observed for model solution No. 2, because DOS is a weak acid.

For model solution No. 4, the effects of organic and inorganic additives are similar, but not additive. These additives compete with each other.

### Influence of calcium and aluminum ions on the formation and stability of colloid iron compounds in the model solutions

As shown earlier (Serikov et al. 2009), stable iron colloid substances are formed in waters with a low hardness salt concentration. The groundwater hardness depends more on calcium salt than on magnesium salt (Serikov et al. 2009). Moreover, no aluminum ions are found in groundwater where iron colloid substances are formed. Therefore, model solutions were used to study the effect of calcium and aluminum ions on the formation and stability of colloid iron substances. The pH was 10.0 ± 0.2.

In experiments, calcium chloride (2.5 · 10^-4^ M) and aluminum chloride (5 · 10^-4^ М) solutions were used. This concentration corresponded to the hardness salt concentration in the groundwater of the Western Siberian region. The *ζ-*potential and the particle size in the model solutions were studied after addition of calcium and aluminum ions. The properties of iron colloid substances depend on the calcium and aluminum ion concentration in the model solution.

Table 5 presents properties of the dispersed phase in model solution No. 2 after addition of calcium ions 5 · 10^-4^ М (20.0 ± 3.5 mg/L) and aluminum ions 2.1 · 10^-6^ М (0.50 ± 0.02 mg/L).

**Table 5:** **Properties of the dispersed phase in model solution No. 2 added with calcium and aluminum ions**

Iron ion concentration, mg/L	DOS concentration, mg/L	Calcium ion concentration, mg/L	Aluminum ion concentration, mg/L	Average particle size, nm	ζ-potential, mV
5.6	4	0	0	99	-42
		20	0	1915	-20
		0	0.5	1950	-18

The data in Table 5 shows that the addition of calcium and aluminum ions results in aggregates of size larger than 1 μm. The *ζ-*potential is close to zero and the iron hydroxide (III) precipitates.

The addition of aluminum and calcium ions increases the particle size to more than 1 μm and decreases the *ζ-*potential. Colloid iron substances precipitate in model solution No. 3 at an iron to silicon ion ratio of 5.6:20 mg/L. The results are presented in Table 6.

**Table 6:** **Properties of the dispersed phase in model solution No. 3 added with calcium and aluminum ions**

Iron ion concentration, mg/L	Silicon ion concentration, mg/L	Calcium ion concentration, mg/L	Aluminum ion concentration, mg/L	Average particle size, nm	ζ-potential, mV
5.6	20	0	0	175	-38
		20	0	1200	-29
		0	0.5	1285	-27

The addition of calcium and aluminum ions to model solution No. 4 with the same concentration as that in solution No. 3 less affects the formation of colloid iron particles, compared to solutions No. 2 and 3.

Table 7 presents the *ζ*-potential and the particle size in model solution No. 4 after addition of calcium and aluminum ions.

**Table 7:** **Properties of the dispersed phase in model solution No. 4 added with calcium and aluminum ions**

Iron ion concentration, mg/L	DOS concentration, mg/L	Silicon ion concentration, mg/L	Calcium ion concentration, mg/L	Aluminum ion concentration, mg/L	Average particle size, nm	ζ-potential, mV
5.6	4	20	0	0	84	-30
			20	0	480	-28
			0	0.5	540	-25

The data in Table 7 demonstrate that the size of formed colloid particles is smaller than 480 and 540 nm when model solution No. 4 is added with calcium and aluminum ions, respectively. It is likely that the amount of calcium ions (at a concentration of 20 mg/L) and aluminum ions (at a concentration of 0.5 mg/L) added to the system is insufficient to compensate negatively charged colloid particles and to form a Fe(OH)_3_ sediment.

**Figure 1:**
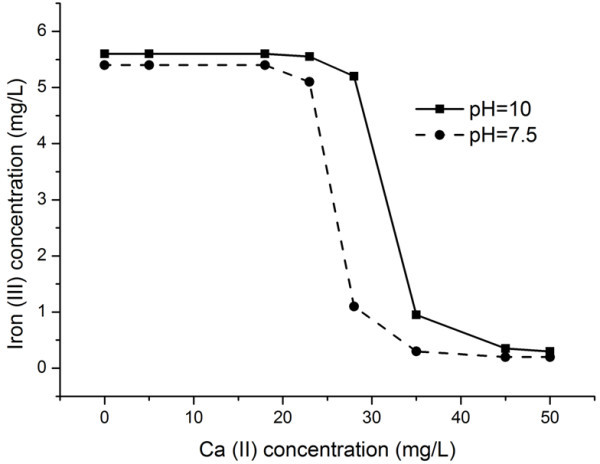
**Iron concentration vs. the calcium concentration in model solution No. 3.**

**Figure 2:**
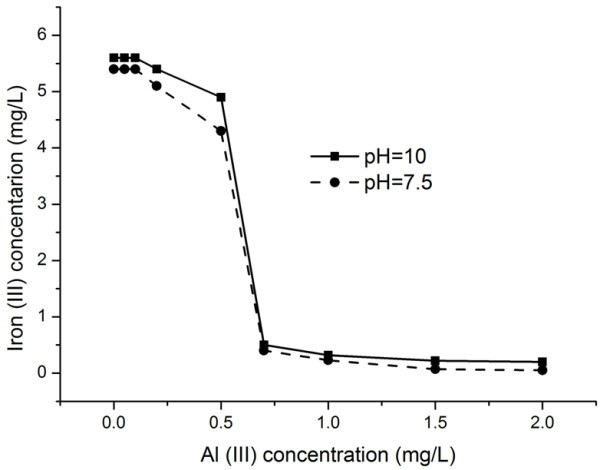
**Iron concentration vs. the aluminum concentration in model solution No. 4.**

The size particles effect in coagulation mechanism shows that the calcium and aluminum ions diffuse to the electric double layer of iron sol and recharge potential determining ions. The iron sol begins to aggregate accompanied with an increase in the size of particles.As the calcium and aluminum ion concentration is decreased, particles of size larger than 1 μm are formed. Figures 1 and 2 show dependences of the iron ion concentration on the calcium and aluminum ion concentrations at a рН of 10.0 ± 0.2 and 7.5 ± 0.2 and constant concentration of silicate ions in solution No. 4.

It is seen in Figure 1 that coagulation of Fe(OH)_3_ begins at a calcium ion concentration of ~25 mg/L. This concentration is considered sufficient for coagulation and sedimentation. It is also seen in the figure that the calcium ion concentration for the solution with pH = 7.5 is less than that for the solution with pH = 10 because the solution with pH = 10 is more stable than the solution with pH = 7.5.

Figure 2 demonstrates that precipitation of iron in model solution No. 4 begins at an aluminum concentration of 0.5 mg/L. The diagram shows that the formation of Fe(OH)_3_ sediment depends weakly on the pH of the solution when the aluminum concentration in the solution is increased. Unlike calcium ions, aluminum ions form polynuclear hydroxycomplexes in water, producing a stronger effect on coagulation. The fact is confirmed by the onset of colloid iron precipitation at an aluminum ion concentration of 0.5.

The results obtained for the model solutions agree well with those for groundwater. Surely, colloid iron is not formed in groundwater containing calcium ions at concentrations above 50 mg/L and aluminum ions. The iron-containing colloids are formed in the Western Siberia groundwater containing iron (II) ions, organic substances, and silicon at a low hardness salt content (less than 25 · 10^-4^ М). These colloid systems are highly resistant to physical and chemical effects, which is used in advanced water purification technologies. Taking into account that the dispersed phase size in the groundwater is about 400 nm, it is necessary to increase the efficiency of filtering elements in reagentless technological processes.

## Conclusions

1. Experimental modeling was performed to study the chemical properties of colloid solutions containing iron ions, dissolved organic substances, and silicon ions. Dissolved organic substances and silicate ions increase the stability of iron sols and complicate water purification from them.
2. Hardness salts and aluminum ions in groundwater prevent the formation of iron colloid solutions and decrease coagulation stability of iron sols due to the recharging iron sols with calcium and aluminum ions.
3. It is found that at a calcium to silicon ratio of 3:1 (6 · 10^-4^ mol: 2 · 10^-4^ mol), particles of size more than 1 μm are formed. These particles are capable for coagulation and sediment formation.
4. The aluminum concentration (0.5 mg/L) may indicate as the minimum effective coagulant dose for the water treatment of the groundwater in Northern regions of Western Siberia.
